# DUX4 Differentially Regulates Transcriptomes of Human Rhabdomyosarcoma and Mouse C2C12 Cells

**DOI:** 10.1371/journal.pone.0064691

**Published:** 2013-05-22

**Authors:** Vishakha Sharma, Naoe Harafuji, Alexandra Belayew, Yi-Wen Chen

**Affiliations:** 1 Department of Molecular Medicine, George Washington University, Washington DC, United States of America; 2 Center for Genetic Medicine Research, Children's National Medical Center, Washington DC, United States of America; 3 Laboratory of Molecular Biology, University of Mons, Mons, Belgium; 4 Department of Integrative Systems Biology, George Washington University, Washington DC, United States of America; Florida State University, United States of America

## Abstract

Facioscapulohumeral muscular dystrophy (FSHD) is linked to the deletion of the D4Z4 arrays at chromosome 4q35. Recent studies suggested that aberrant expression of double homeobox 4 (*DUX4*) from the last D4Z4 repeat causes FSHD. The aim of this study is to determine transcriptomic responses to ectopically expressed DUX4 in human and mouse cells of muscle lineage. We expression profiled human rhabdomyosarcoma (RD) cells and mouse C2C12 cells transfected with expression vectors of *DUX4* using the Affymetrix Human Genome U133 Plus 2.0 Arrays and Mouse Genome 430 2.0 Arrays, respectively. A total of 2267 and 150 transcripts were identified to be differentially expressed in the RD and C2C12 cells, respectively. Amongst the transcripts differentially expressed in the RD cells, *MYOD* and *MYOG* (2 fold, p<0.05), and six *MYOD* downstream targets were up-regulated in RD but not C2C12 cells. Furthermore, 13 transcripts involved in germline function were dramatically induced only in the RD cells expressing DUX4. The top 3 IPA canonical pathways affected by DUX4 were different between the RD (inflammation, BMP signaling and NRF-2 mediated oxidative stress) and the C2C12 cells (p53 signaling, cell cycle regulation and cellular energy metabolism). Amongst the 40 transcripts shared by the RD and C2C12 cells, *UTS2* was significantly induced by 76 fold and 224 fold in the RD and C2C12 cells, respectively. The differential expression of *MYOD, MYOG* and *UTS2* were validated using real-time quantitative RT-PCR. We further validated the differentially expressed genes in immortalized FSHD myoblasts and showed up-regulation of *MYOD*, *MYOG*, *ZSCAN4* and *UTS2*. The results suggest that DUX4 regulates overlapped and distinct groups of genes and pathways in human and mouse cells as evident by the selective up-regulation of genes involved in myogenesis and gametogenesis in human RD and immortalized cells as well as the different molecular pathways identified in the cells.

## Introduction

FSHD is an autosomal dominant disorder and the third most common inherited form of muscular dystrophy. The disease is characterized by a progressive and selective weakness and atrophy of the facial, scapular, and humeral muscles followed by weakness of muscles of the lower extremities. The weakness of muscles is often asymmetric. There are currently no pharmacologic therapies available to treat this disease [1]–[4]. FSHD1 (OMIM #158900) affects 95% of patients and is genetically linked to contractions of the D4Z4 repeat array at chromosome 4q35 from 11–150 repeat units in healthy individuals to 1–10 repeat units in patients with FSHD. Individuals without any repeat do not develop FSHD [1]–[4]. Each of the repeat units contains a conserved ORF for the double homeobox 4 (*DUX4*) gene, which is aberrantly transcribed from the last repeat in patients. FSHD2 (OMIM #158901) is not linked to contractions of the D4Z4 repeat array but to mutations in the SMCHD1 protein involved in chromatin structure [5]. DNA hypomethylation of the D4Z4 region is common to both FSHD1 and 2 and causes transcriptional de-repression, which allows the *DUX4* gene to be transcribed. The FSHD permissive alleles further present a poly-adenylation signal in the pLAM region distal to the repeat array [6], which allows stabilization of the *DUX4* transcripts derived from the last D4Z4 unit and their translation [6]–[14].

The DUX4 protein is a homeodomain transcription factor [6], [9]. The function of DUX4 has been primarily studied using mice [15], [16]. Previous studies showed that ectopic expression of human DUX4 in C2C12 cells induced genes involved in oxidative stress as well as suppressed MYOD pathways [15]. In addition, ectopic DUX4 expression induced p53-dependant muscle cell death both *in vitro* and *in vivo* [15], [16]. While some of the findings in these mouse studies agree with what has been reported in studies of human muscle biopsies and myoblasts, including the involvement of oxidative stress responses and cell apoptosis, others such as suppression of MYOD signaling did not agree with findings using patient samples [17]–[26]. Considering the differences between the mouse and human studies, and the fact that there is no orthologue of DUX4 in the mouse genome although two paralogues were reported [8], it is critical to know whether the transcription regulatory targets of human DUX4 are the same in mouse cells. The knowledge will allow us to determine whether DUX4-regulated pathways can be properly studied in mouse models.

In this study, we compared transcriptomic changes that are induced in response to ectopic DUX4 expression in human and mouse cell lines of muscle lineage. Expression profiling studies of human RD cells and mouse C2C12 cells transfected with *DUX4* expression vectors were conducted. The C2C12 cell line is a mouse myoblast cell line that derived from skeletal muscles of C3H mice [27] and has since been commonly used to study cellular and molecular pathways in muscle [6], [15]. The human RD cell line is a rhabdomyosarcoma cell line that was derived from a human embryonal rhabdomyosarcoma [28]. This cell line expresses myogenic markers and has been used extensively for studying regulatory pathways in muscles [29], [30]. In this study the mRNA expression changes of the RD and C2C12 cells in response to ectopic DUX4 expression was studied and compared. Considering the RD cells are of neoplastic origin, we also validated our results using immortalized human myoblasts from patients with FSHD.

## Methods

### Cell Culture and Transfection

The cell culture and transfection experiments of both the RD from American Type Culture Collection (ATCC) and C2C12 cells (ATCC) were conducted in parallel under the same conditions. A total of 1×10^5^ cells were seeded and cultured to 60% confluence in Dulbecco's modified Eagle's medium containing 10% heat inactivated fetal bovine serum (Sigma-Aldrich) and 1% penicillin-streptomycin in 25 cm^2^ flasks at 37°C, 5% CO2. The cells were transfected with 6.25 µg pCIneo-DUX4 [9] expression vector (n = 4) using Lipofectamine LTX (Life Technologies) according to the manufacturer's protocol, and cells collected 16 hours afterwards. Cells transfected with pCIneo insertless vector were used as controls. Transfection efficiency was determined using cells transfected with GFP expression vector. Percentages of GFP positive cells of 5 random fields were calculated and averaged. The transfection efficiency in C2C12 and RD cells were 91% (±3%) and 89% (±1%), respectively.

Immortalized human myoblasts were obtained from the Senator Paul Wellstone Muscular Dystrophy Cooperative Research Center at Boston Biomedical Research Institute. The patient myoblast cell line was derived from the biceps of a 42 years old male with mild muscle weakness (WS157) [31]. The control myoblasts were derived from the patient's 46 year old brother without FSHD (WS161) [31]. These cells were immortalized with expression vectors encoding hTERT that compensates for telomere loss and CDK4 that prevents growth arrest of CD56+ myogenic cells, when these cells are cultured *in-vitro*. We cultured these cells as described in previously published protocol [31], [32]. Briefly, proliferating immortalized myoblasts were cultured in a growth medium consisting of medium 199 and DMEM (Life Technologies) in a 1∶4 ratio with 0.8 mM sodium pyruvate (Life Technologies), 3.4 g/l sodium bicarbonate (Sigma-Aldrich), 15 % fetal bovine serum (Thermo Scientific), 0.03 µg/ml Zinc sulfate (Fisher), 1.4 µg/ml vitamin B12 (Sigma-Aldrich), 2.5 ng/ml recombinant human hepatocyte growth factor (Millipore), 10 ng/ml basic fibroblast growth factor (Biopioneer), 0.02 M HEPES, and (Life Technologies) at 37°C, 5% CO2. The culture dish was coated with 0.1 % gelatin (Sigma-Aldrich).

### Expression Profiling and Data Analyses

Affymetrix Human Genome U133 Plus 2.0 and Mouse 430 Plus 2.0 arrays were used for profiling the RD and C2C12 cells, respectively. The procedures were conducted as previously described [6]. Briefly, total RNA was isolated from human RD and mouse C2C12 cells using TRIzol (Invitrogen) according to the manufacturer's protocol and purified using the RNeasy MinElute Cleanup Kit (Qiagen) according to the manufacturer's protocol. Four hundred nanograms of total RNA was converted into double stranded cDNA, then biotin labeled cRNA, which was subsequently fragmented. All of the steps were performed using the Affymetrix 3′-IVT Express Kit. The fragmented cRNA was hybridized to microarrays for 16 hours at 45°C. Following hybridization, the washing and staining steps were performed using Fluidics Station 450 as described in the Affymetrix protocol. The probe arrays were subsequently scanned using the Genechip Scanner 3000 to acquire images providing the raw data of gene expression. The microarray data generated is deposited to the Gene Expression Omnibus (GEO) database (accession number GSE45854).

The raw data were imported into GeneSpring GX 11.0 software (Silicon Genetics, CA, USA) for filtering and statistical analysis. The probe sets showing at least one Affymetrix ‘present’ calls out of a total of eight human or mouse arrays (∼10% Present calls), respectively, were selected for further statistical analysis. Welch's *t* test was performed to calculate the probabilities of significant gene expression changes (p<0.05) along with multiple testing correction using Benjamini Hochberg False Discovery Rate (5%).

The gene lists generated in Genespring were imported into Ingenuity Pathway Analysis (IPA, Ingenuity Systems, Redwood, CA), which is a web based bioinformatics tool used to identify canonical pathways differentially regulated in microarray datasets. The significance of genes from the microarray data assigned to pathways by IPA is determined by the ratio of the number of genes in the dataset mapping to a specific pathway to the total number of genes in the IPA database mapping to that pathway. Fischer's exact test was used to calculate a p-value that determines whether the association between the gene and the pathway is significant. The pathways are subsequently ranked according to the p-value.

### Real-time Quantitative Reverse Transcription Polymerase Chain Reaction (real-time qRT-PCR)

Real-time qRT-PCR was performed to validate microarray results as previously described [6], [33]. Briefly, total RNA (1 µg) from each sample was first subjected to DNAse I digestion (1 U) in 1× DNAse I reaction buffer (Promega) by incubating at 37°C for 30 minutes to remove genomic DNA contamination. The reaction was inactivated by adding 1 µl of stop solution (Promega) and heating for 10 minutes at 65°C. Subsequently, the RNA sample was reverse transcribed to cDNA using Superscript II (Life Technologies) and oligo dT primers. The cDNA thus generated was amplified in triplicates in SYBR Green PCR Master Mix (Life Technologies) using 1 µM of forward and reverse primers specific to each gene and 1 µl of cDNA template in a total volume of 50 µl. The thermal cycling conditions included 50°C for 2 min, 95°C for 10 min, followed by 40 cycles of amplification using the condition of 95°C for 15 s then 60°C for 1 min. Primer sequences used for human *myogenic differentiation 1* (*MYOD*) were (forward) 5′ -TGCTCCGACGGCATGATGGAC -3′ and (reverse) 5′-TCGACACCGCCGCACTCT -3′; *urotensin 2* (*UTS2*) were (forward) 5′- AAGTTTCAGGATTTCTCTGGACAAGATCC -3′ and (reverse) 5′- CCAGAAGCAATCAGGAGTCTCACG-3′; *myogenin* (*MYOG*) (forward) were 5′-AACCCAGGGGATCATCTGCTCAC-3′ and (reverse) 5′-GTTGGGCATGGTTTCATCTGGGAAG-3′; *zinc finger and SCAN domain containing 4* (*ZSCAN4*) were (forward) 5′-TGGAAATCAAGTGGCAAAAA-3′ and (reverse) 5′-CTGCATGTGGACGTGGAC-3′ [24]. *Glyceraldehyde-3-phosphate dehydrogenase* (*GAPDH*) was used as internal control and the primers used were (forward) 5′- TGTCAAGCTCATTTCCTGGTA-3′ and (reverse) 5′- GTGAGGGTCTCTCTCTTCCTCTTGT-3′. Primer sequences used for mouse *urotensin 2* (*Uts2*) were (forward) 5′-GAGGAAGGCTTTCGCTGGGCA-3′ and 5′-GGGCAGCCCCGTGTTGCTTA-3′. *Glyceraldehyde-3-phosphate dehydrogenase* (*Gapdh*) was used as internal control and the primers used were (forward) 5′-CCAGGAGCGAGACCCCACTAACA-3′ and (reverse) 5′-TCAAGTGGGCCCCGGCCTT-3′. All primers were tested for nonspecific amplicons and primer dimers by visualizing PCR products on 1% agarose gels as well as melting curve analysis. The ΔΔCT method was used to determine expression values relative to *GAPDH* as well as fold differences relative to insertless vector. T-test was used (*P*<0.05) to determine statistical significance.

## Results

### DUX4 regulates distinct groups of transcripts in the RD cells

To determine the molecular responses to ectopic expression of DUX4 in human RD and mouse C2C12 cells, we expression profiled human RD and mouse C2C12 cells transfected with *DUX4* expression vector. The cells were cultured and transfected in parallel and same statistical criteria were applied when the array data were analyzed. A total of 2267 transcripts were differentially expressed in RD cells (Table S1), while 150 differentially expressed transcripts were identified in C2C12 cells (Table S2). A total of 40 differentially expressed transcripts were shared between the two cell lines (Table S3). Among the shared genes, the direction of expression changes of the majority of the genes were the same suggesting these responses were truly shared between the RD and C2C12 cells. Molecular pathways affected by DUX4 in human RD cells and mouse C2C12 cells were further examined using Ingenuity Pathway Analysis (IPA). The results showed that top 3 canonical pathways affected by DUX4 expression in RD cells were those involved in Wnt-mediated immune responses, BMP signaling and NRF-2 mediated oxidative stress response, whereas in C2C12 cells were those involved in p53 signaling, cell cycle regulation, and cellular energy metabolism. The results showed that the most significantly affected molecular pathways by DUX4 are distinct in the RD cells and C2C12 cells while some expression changes were shared.

The Wnt-mediated inflammatory immune response pathway was found to be the top ranked pathway affected by DUX4 in the RD cells as evidenced by significant up-regulation of *WNT5A* (1.4 fold, p<0.05), and several frizzled family receptors, namely *FZD1* (1.6 fold, p<0.01), *FZD2* (1.5 fold, p<0.05), *FZD4* (1.5 fold, p<0.01), and *FZD7c* (1.6 fold, p<0.01) (Table S4). However, the transcripts of interleukins *IL6* (−1.8 fold, p<0.05), *IL8* (−2.2 fold, p<0.01), and *IL15* (−1.4 fold, p<0.05), which are downstream of WNT5A were down-regulated.

The BMP signaling pathway was found to be the second ranked pathway. Of the 39 transcripts that belong to this pathway in the IPA database, 17 transcripts were misregulated in response to ectopic DUX4 expression in the RD cells. Amongst the misregulated transcripts, 53% of the expression changes were shown to be involved in suppression of the BMP signaling pathway while 35% of the changes indicated activation of the pathway (Table S5).

The NRF-2 mediated oxidative stress response pathway was identified to be the third ranked pathway regulated by DUX4 in the RD cells. Thirty one of the 86 transcripts known to function in this pathway were found to be misregulated in this study. Among the 31 differentially expressed transcripts, 55% of the changes may potentially induce or contribute to oxidative stress, while 39% of them were reported to be involved in anti-oxidative stress responses (Table S6).

### DUX4 significantly up-regulated genes involved in myogenesis and gametogenesis in the RD cells but not C2C12 cells

**Figure 1:**
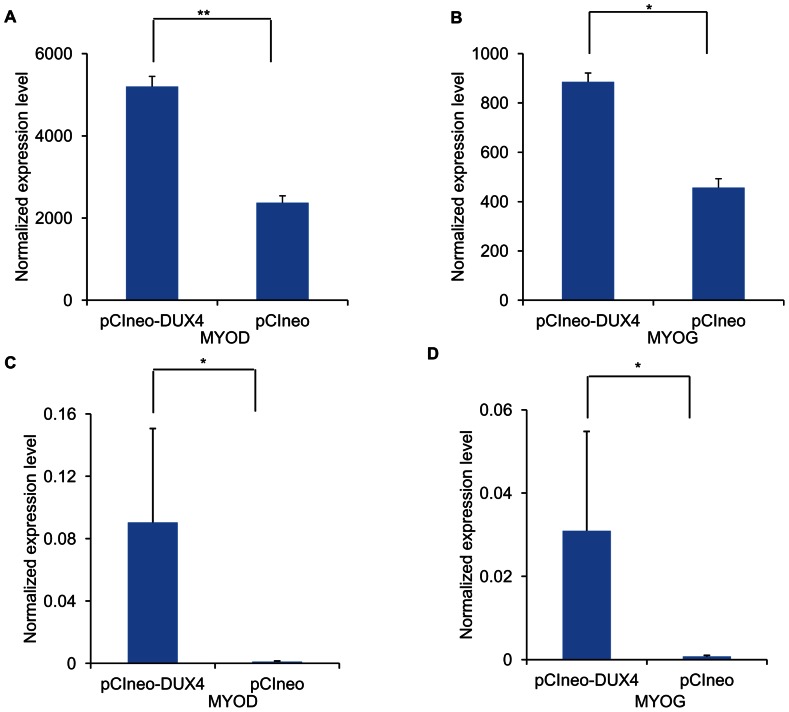
Up-regulation of *MYOD* and *MYOG* in response to ectopic DUX4 expression in RD cells. Expression levels of myogenic markers *MYOD* (A) and *MYOG* (B) were determined by expression profiling human RD cells transfected with an expression vector either encoding DUX4 or insertless (control), respectively. The differential expression of *MYOD* (C) and *MYOG* (D) were validated using real-time qRT-PCR (n = 4). Normalized expression levels of the transcripts were calculated using *GAPDH* as a reference. ** p<0.01, * p <0.05.

Since myogenesis factors and genes regulating cell cycle have previously been reported to be affected in primary FSHD myoblasts [19], [21], [26], we first looked up the expression changes of two major myogenic factors, *MYOD* and *MYOG*, in the profiling data. The results showed that *MYOD* (Figure 1A) and *MYOG* (Figure 1B) were 2 fold up-regulated in the RD cells (p<0.05) but not in the C2C12 cells ectopically expressing DUX4. Six transcripts reported as direct targets of MYOD, namely *BIN1* (1.4 fold, p<0.05), *HMGB3* (1.3 fold, p<0.05), *SIX1* (1.5 fold, p<0.05), *ACTC1* (1.3 fold, p<0.05), *IGFBP5* (1.5 fold, p<0.05), and *CHRNA1* (2.1 fold, p<0.05) [34], were also up-regulated only in the RD cells transfected with the *DUX4* expression vector. In addition, several transcripts involved in cell cycle progression were shown down-regulated in the RD cells, including *CCND1* (−1.3 fold, p<0.05), *CCND2* (−1.3 fold, p<0.05), *CDC6* (−1.3 fold, p<0.01) and *E2F7* (−1.5-fold, p<0.05) but not in the C2C12 cells. To validate the significant up-regulation of *MYOD* and *MYOG* in the RD cells, we performed real-time qRT-PCR and confirmed that both *MYOD* (20 fold, p<0.05) and *MYOG* (12 fold, p<0.05) were up-regulated in the RD cells ectopically expressing DUX4 (Figure 1C–D).

**Figure 2:**
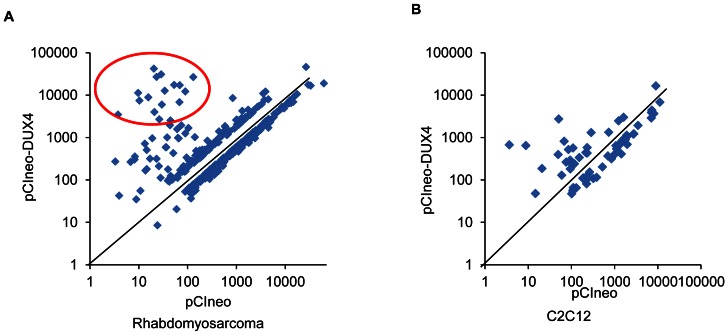
Scatter plot analysis of transcripts regulated by ectopically expressed DUX4 in RD and C2C12 cells. To clearly visualize transcripts highly induced by DUX4, only transcripts changed >2-fold were used for analysis. Log transformed expression levels of transcripts in cells transfected with the insertless vector was plotted against expression levels of transcripts in cells transfected with the *DUX4* expression vector. A cluster of transcripts highly induced by DUX4 (circled) in RD (A.) but not C2C12 cells (B.) was observed.

A small number of genes showed dramatic up-regulation (>100-fold) only in the RD cells ectopically expressing DUX4, which formed a cluster of transcripts (Figure 2A–B). These transcripts were *MBD3L2* (2805 fold, p<0.01), *TRIM43* (2060 fold, p<0.01), *ZSCAN4* (1546 fold, p<0.01), *RFPL1/RFPL2* (1231 fold, p<0.01), *PRAMEF1/PRAMEF13/PRAMEF2* (1217 fold, p<0.01), *PRAMEF12* (711 fold, p<0.01), *TRIM48* (421 fold, p<0.01), *TRIM49* (409 fold, p<0.05), *RFPL2* (332 fold, p<0.01), *KHDC1L* (254 fold, p<0.01), *RFPL3* (204 fold, p<0.01), *SPRYD5* (171 fold, p<0.01), and *PRAMEF11* (126 fold, p<0.01). Out of these, only *Zscan4* was up-regulated (7.4 fold, p<0.01) in C2C12 cells ectopically expressing DUX4. While not expressing in normal skeletal muscle, these transcripts are expressed in germ cells, embryos during preimplantation and early embryogenesis. The genes were also reported to be up-regulated in immortalized human myoblasts ectopically expressing DUX4 [20].

### DUX4 induced up-regulation of UTS2 in both the RD and C2C12 cells ectopically expressing DUX4

**Figure 3:**
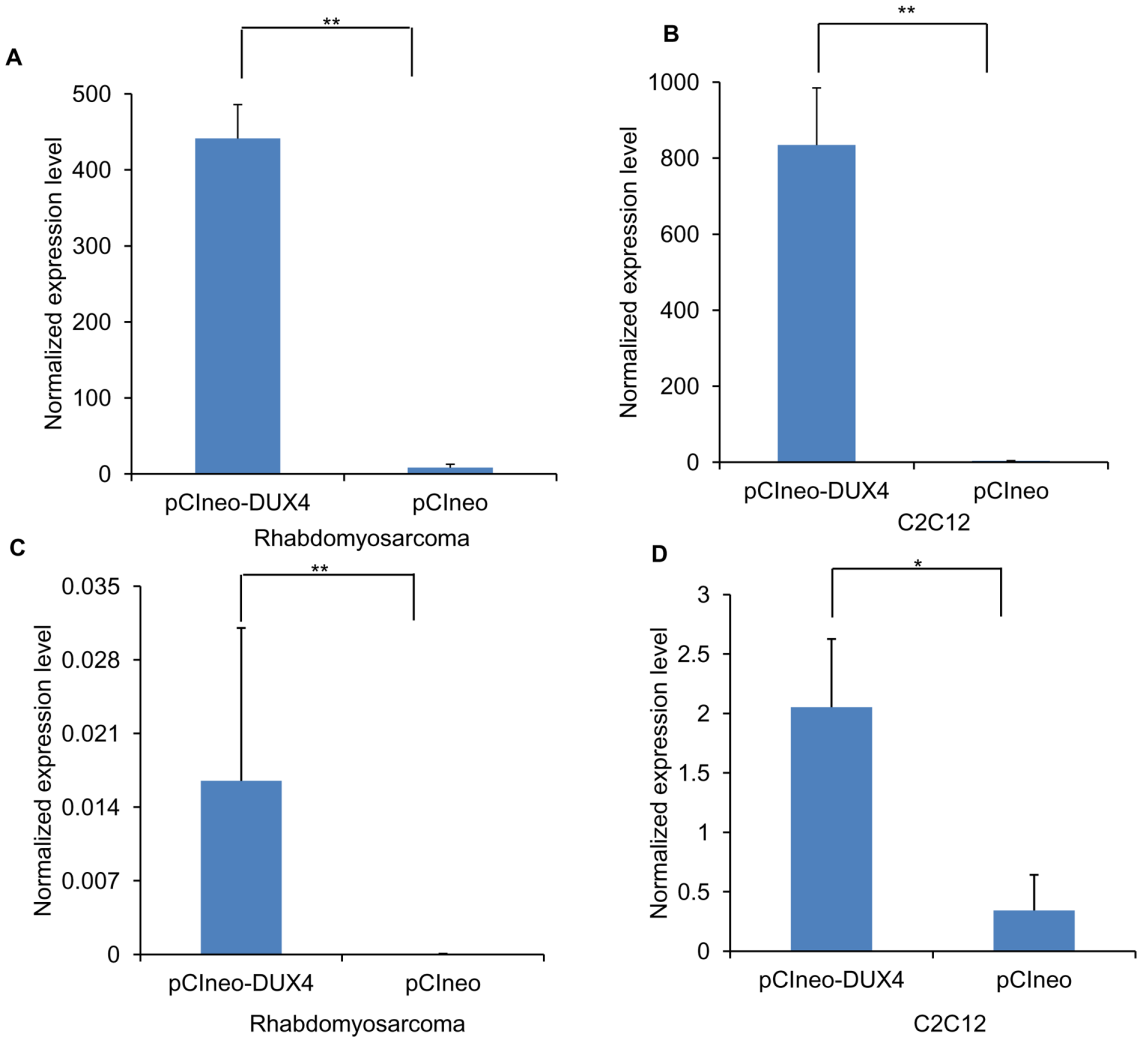
Up-regulation of human UTS2 and mouse Uts2 was observed in RD and C2C12 cells ectopically expressing DUX4, respectively. Expression levels of human *UTS2* and mouse *Uts2* were determined by expression profiling RD (A) and C2C12 (B) cells transfected with an expression vector either encoding DUX4 or insertless (control), respectively. The expression changes in RD (C) and C2C12 (D) were validated using real-time qRT-PCR (n = 4). Normalized expression levels of the transcripts were calculated using *GAPDH* and *Gapdh* as a reference in both cell lines. ** p<0.01, * p <0.05.

While the majority of the genes induced by DUX4 expression in RD and C2C12 cells were cell-specific, 40 transcripts were similarly regulated by DUX4 in both cell lines suggesting there are shared transcriptional targets in the cells (Table S3) and urotensin-2 (*UTS2*) was one of them. *UTS2* was significantly up-regulated in the RD cells (76 fold, p<0.01) and C2C12 cells (224 fold, p<0.01) ectopically expressing DUX4 (Figures 3A–B). The finding were validated by real-time qRT-PCR in both the RD and C2C12 cells with fold changes of 130 fold (p<0.01) and 21 fold (p<0.05) respectively (Figure 3C–D).

### Transcripts upregulated by ectopic DUX4 expression were upregulated in immortalized FSHD myoblasts

**Figure 4:**
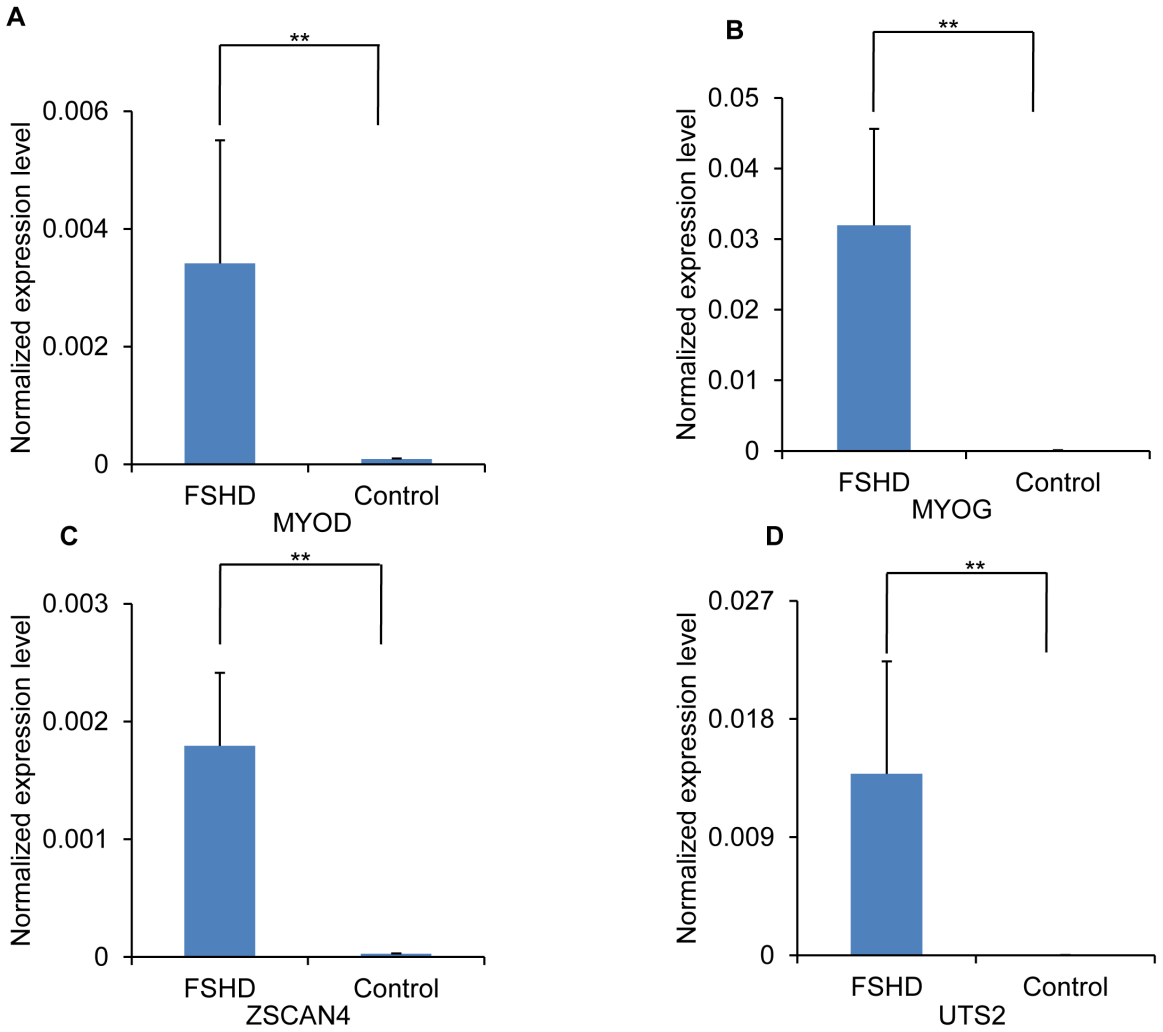
Up-regulation of *MYOD*, *MYOG*, *ZSCAN4*, and *UTS2* in FSHD immortalized cells. *MYOD* (A.), *MYOG* (B.), *ZSCAN4* (C.), and *UTS2* (D.) levels were quantified in FSHD immortalized cells and control cells using real-time qRT-PCR (n = 4). Values representing expression levels of transcripts were calculated using *GAPDH* as a reference. ** p<0.01.

To determine whether the expression changes identified in the RD cells ectopically expressing DUX4 can be detected in the FSHD myoblasts, we performed real-time qRT-PCR and validated the significant up-regulation of *MYOD* (19 fold, p<0.01), *MYOG* (110 fold, p<0.01), *ZSCAN4* (32 fold, p<0.01), and *UTS2* (229 fold, p<0.01) (Figure 4A–D) in immortalized FSHD cells as compared to the control immortalized myoblasts.

## Discussion

Misregulation of genes and pathways involved in myogenesis, cell cycle regulation and oxidative stress in human FSHD myoblasts has been reported previously [13]–[23]. Recently genes involved in gametogenesis were shown to be up-regulated in immortalized FSHD myoblasts and muscle biopsies [20], [35], [36]. While some of the changes were also reported in studies conducted using C2C12 cells and animal models, such as increased susceptibility to oxidative stress and the induction of cell death, other findings did not agree with the human studies including how the myogenesis program was affected by DUX4 and the induction of the germline genes [15], [16], [35], [36]. To further investigate the genes and pathways regulated by DUX4 in human and mouse cells of muscle lineage, we analyzed mRNA transcripts affected by ectopically expressed DUX4 using expression profiling. Our results showed that while the RD cells expressing DUX4 recapitulated the molecular defects seen in human muscles and immortalized myoblasts, the C2C12 cells responded differently to the DUX4 expression. For example, the most dramatic expression changes of the germline genes were induced by DUX4 in both RD and immortalized FSHD myoblasts and observed in patients' muscles but not the C2C12 cells. Among these genes, only ZSCAN4 was mildly up-regulated in the C2C12 cells. In general, DUX4 regulated significantly greater number of genes (2267) in RD cells as compared with C2C12 cells (150) suggesting that DUX4 may have more direct regulatory targets in RD cells as compared with C2C12 cells. In addition, several pathways and genes previously reported to be misregulated in FSHD were shown affected in the RD cells but not in the C2C12 cells. The similarity among the human RD cells, the immortalized FSHD myoblasts and the primary myoblasts suggest that RD and immortalized FHSD myoblasts may be more suitable culture systems for studying DUX4 function than mouse C2C12 cells. Whether mouse or other animal models that carry human *DUX4* in their genomes can be used to study DUX4 function or can be a suitable FSHD disease model need to be further investigated.

Previous studies showed up-regulation of MYOD and its downstream regulatory targets accompanied by a halt in cell cycle progression in human myoblasts and biopsies therefore a hypothesis that pre-mature activation of myogenesis program was involved in the pathological mechanisms of FSHD was proposed [19], [21], [26]. MYOD is a transcription factor that activates myogenesis through regulation of several transcriptional targets that facilitates the transformation of quiescent satellite cells (stem cells committed to muscle lineage) into proliferating myoblasts which are capable of undergoing differentiation [37]. While the activated myoblasts are essential for muscle maintenance and repair, maintaining a healthy number of satellite cells is critical for a continuous supply of myoblasts [38]. A pre-mature activation of myogenesis induced by DUX4 bares a risk to prematurely deplete satellite cells, which can potentially lead to diminished regenerative capacity of the adult skeletal muscle. Our profiling and real-time qRT-PCR data showed up-regulation of *MYOD* and *MYOG* as well as suppression of cell cycle progression in the RD cells expressing DUX4. The up-regulation of *MYOD* and *MYOG* was further validated using immortalized FHSD myoblasts. The findings are in concordance with previous studies in primary FSHD myoblasts and muscle biopsies [19], [21], [26]. Our data provide a direct link between DUX4 expression and the activation of myogenesis program as evidenced by activation of *MYOD* and its downstream target genes accompanied by a halt in cell cycle progression, a critical step prior to differentiation.

A recent study by Geng et al [20] showed activation of *MYOG* expression in response to ectopic expression of DUX4 in human immortalized myoblasts, which is consistent with activation of MYOD signaling since terminal differentiation involves increased MYOG and decreased MYOD expression levels, a step necessary for myoblasts to fuse and form mature myofibers [39], [40]. Since our results showed higher expression levels of both *MYOD* and *MYOG* by DUX4 overexpression, this indicates these cells are at an earlier stage of differentiation process. This could be explained by the fact that our study was conducted at an earlier time point (16 hours post-transfection) as compared to the study conducted by Geng et al (24 hours post-transfection). While the studies conducted using human muscle biopsies and myoblasts reported activation of MYOD pathways [19], [21], [26], studies conducted in C2C12 cells reported repression of MYOD pathways in response to ectopically expressed DUX4 as well as DUX4c [15], [41] The different conclusions from human and mouse studies can potentially be explained by the lack of *DUX4* orthologue in mice; therefore human DUX4 does not regulate *Myod* and other regulatory target genes the same way.

In addition to activation of MYOD program, we also identified a suppression of BMP signaling pathways in RD cells expressing DUX4. The BMP pathways negatively regulate MYOD program and was identified as the second top ranked pathway in the RD cells ectopically expressing DUX4. BMP signaling has been shown to be activated during the proliferation stage of satellite cells. The BMP signaling is suppressed during differentiation by a BMP antagonist Noggin [42]. Our profiling data suggested a suppression of the BMP signaling with the BMP antagonist *Noggin* up-regulated by DUX4 (1.6 fold, p<0.05; Table S5). Overall, the data support our conclusions that DUX4 expression leads to induction of myogenesis through activation of MYOD signaling and increased expression of Noggin.

The top ranked pathway identified by IPA to be differentially regulated in RD cells ectopically expressing DUX4 are genes involved in innate immune response as evidenced by up-regulation of *WNT5A*, and several *FZD* receptors. Genomic studies have shown *WNT5A*, agonist of *FZD* receptors, to be up-regulated in T-cells, macrophages and dendritic cells exposed to pathogens as well as in pathologies involving inflammation such as rheumatoid arthritis. Previous studies suggest that Wnt5A is a positive regulator of immunity and inflammation [43]–[47]. Our data therefore indicate that DUX4 directly induces an inflammatory immune response, which could contribute to the T-cell mediated inflammation in FSHD reported previously [35], [48]. Interestingly, some interleukin genes functioning in lymphocyte activation and infiltration downstream of Wnt5A are downregulated in our RD data suggesting compensatory mechanisms to combat Wnt5A signaling. However, while WNT5A can be involved in regulating immune responses, it is also highly expressed in satellite cells and is involved in switching cells from proliferation to myogenic differentiation [49]. The up-regulation of Wnt signaling in the myoblasts can potentially contribute to increased myogenesis and not related to inflammation.

The top ranked pathway affected by DUX4 expression in the C2C12 cells was the p53 pathway. P53 signaling has been shown to be activated by DUX4 in mice ectopically expressing DUX4 *in vivo* and other animal models expressing DUX4 [16], [50]. In addition, it is shown to be up-regulated in human FSHD myoblasts during differentiation [24]. This pathway was also highly ranked among the pathways affected in the RD cells expressing DUX4 but not in the top 3. It should be noted that RD cells are of neoplastic origin and contain point mutations in the tumor suppressing p53 gene leading to its functional loss [51]–[53]. This could potentially explain the reasons for the p53 pathway not being amongst the top 3 ranked affected pathway by DUX4 in RD cells. While the activation of the p53 pathway was suggested based on the IPA, we did not observe obvious reduction of total cell numbers when we collected the cells. In addition, the amount of total RNA isolated from the cells was comparable between the cells transfected with the DUX4 and insertless vectors. We selected an earlier time point to collect cells in order to avoid profiling dying or dead cells. The cell death likely will occur at a later time point.

Previous studies showed that FSHD myoblasts and C2C12 cells expressing DUX4 were more vulnerable to oxidative stress [15], [17]–[19], [23]–[25], [54], while another study of FSHD and control myoblasts from relatives did not [31]. Our data showed that ectopic DUX4 expression in RD cells caused the misregulation of genes involved in the NRF2-mediated oxidative stress response pathway, which is involved in combating oxidative stress. Oxidative stress is caused when the rate of production of reactive oxygen species such as free radicals and peroxides, exceeds their rate of detoxification. Increases in levels of reactive oxygen species can be very damaging to the cell and trigger apoptotic responses. The NRF2 mediated oxidative stress response is the primary pathway involved in combating oxidative stress through the action of several detoxifying and anti-oxidant enzymes functioning in the pathway. Misregulation of transcripts involved in the NRF2 mediated oxidative stress response pathway has been reported in several FSHD studies [15], [17]–[19], [23]–[25], [54]. Our study again confirmed the link with DUX4. In addition, our data showed that more changes that are pro-oxidative stress were induced by DUX4. This pathway was also significant in C2C12 cells expressing DUX4 but was not in top 3.

A novel finding of this study is the dramatic induction of *UTS2* in response to ectopic DUX4 expression in both the RD and C2C12 cells, which was also validated in the immortalized FHSD myoblasts. UTS2 is a powerful vasoconstrictor and has also been shown to be pro-angiogenic as evidenced by its ability to cause increased proliferation of endothelial cells as well as increased migration of vascular smooth muscle cells [55]–[59]. It has also been recently shown associated with diabetic retinopathy and atherosclerosis [60]; therefore, its induction in response to DUX4 provides a potential explanation for the retinal vasculopathy commonly observed in FSHD patients, who are found to exhibit symptoms similar to those exhibited by patients of Coats' disease, wherein abnormal vessels develop behind retina [61], [62]. Since the molecular mechanism of these retinal defects remains yet unknown, the up-regulation of UTS2 could potentially be involved and worth for further investigation.

Interestingly, rhabdomyosarcoma cells have often been used to study pharmacological properties of UTS2 and its receptors since these cells endogenously express UTS2 receptors. Moreover, UTS2 and its receptors have also been shown expressed at a significantly greater level in human skeletal cells and tissues compared with other organs such as pancreas, brain, liver, testis, placenta, lung, kidney, thymus, prostate, small intestine, colon, peripheral blood leukocytes, ovary and spleen. Our findings along with the findings reported in these studies indicate that overexpression of UTS2 in skeletal muscle could be particularly significant in contributing towards the skeletal muscular symptoms in FSHD patients [63]–[66].

Our study suggests that DUX4 can contribute to FSHD pathogenesis through several avenues including induction of MYOD pathways, induction of immune and inflammatory response, misregulation of genes involved in oxidative stress, and induction of germline genes. Our study also reported a dramatic induction of *UTS2*, a potent vasoconstrictor involved in angiogenesis and also reported preferentially expressed in skeletal muscle tissue in FSHD myoblasts, which could potentially explain the vasculopathy and skeletal muscular symptoms observed in FSHD patients. Furthermore, we showed that some of these critical changes were not observed in mouse C2C12 myoblasts while other changes overlapped, which suggest that a mouse model carrying human *DUX4* gene may not fully recapitulate the human FSHD and needs to be evaluated carefully.

## Supporting Information

Table S1 **Transcripts regulated by DUX4 in RD cells.** RD cells transfected with DUX4 expression vector and insertless vector (control) were expression profiled and fold-changes of transcripts changed in response to ectopic DUX4 expression were calculated relative to control. Welch's *t* test was performed to calculate the probabilities of significant gene expression changes (p<0.05) along with multiple testing correction using Benjamini Hochberg False Discovery Rate (5%).
(XLSX)

Table S2 **Transcripts regulated by DUX4 in C2C12 cells.** C2C12 cells transfected with DUX4 expression vector and insertless vector (control) were expression profiled and fold-changes of transcripts changed in response to ectopic DUX4 expression were calculated relative to control. Welch's *t* test was performed to calculate the probabilities of significant gene expression changes (p<0.05) along with multiple testing correction using Benjamini Hochberg False Discovery Rate (5%).
(XLSX)

Table S3 **Common transcripts regulated by DUX4 in RD and C2C12 cells.** Genespring GX 11.0 was used to identify transcripts regulated by DUX4 in both RD and C2C12 cells (p<0.05).
(XLSX)

Table S4 **Transcripts functioning in immune response pathways regulated by DUX4 in RD cells.** Transcripts identified in the top ranked canonical pathway regulated by DUX4 in RD cells were identified through IPA.
(XLSX)

Table S5 **Transcripts functioning in BMP signaling pathway regulated by DUX4 in RD cells.** Transcripts functioning in BMP signaling pathway, the second ranked canonical pathway regulated by DUX4 in RD cells, were identified through IPA.
(XLSX)

Table S6 **Transcripts functioning in NRF2 mediated oxidative stress response pathway regulated by DUX4 in RD cells.** Transcripts functioning in NRF2 mediated oxidative stress response pathway, the third ranked canonical pathway regulated by DUX4 in RD cells, were identified through IPA.
(XLSX)
